# Preparation and Characterization of C-Reactive Protein Dual-Particle Latex-Enhanced Immunoturbidimetric Reagents

**DOI:** 10.34133/bmef.0085

**Published:** 2024-12-23

**Authors:** Yanyan Liu, Meijiao Li, Hao Zhang, Le Gao, Jitao Liu, Xuetong Zhu, Chenzhong Li, Shan Liu, Yue Hou, Jiancheng Xu

**Affiliations:** ^1^ School of Life Science and Technology, Changchun University of Technology, Changchun 130013, China.; ^2^ Jilin Getein Biotechnology Co., Ltd, Changchun, Jilin 130103, China.; ^3^ Department of Laboratory Medicine and Center of Infectious Diseases and Pathogen Biology, The First Hospital of Jilin University, Changchun 130021, China.; ^4^ School of Medicine, The Chinese University of Hong Kong, Shenzhen 518172, China.; ^5^ Sichuan Provincial Key Laboratory for Human Disease Gene Study, Department of Medical Genetics, Sichuan Academy of Medical Sciences & Sichuan Provincial People’s Hospital, University of Electronic Science and Technology of China, Chengdu 610072, China.

## Abstract

**Objective and Impact Statement:** This study aims to couple C-reactive protein (CRP) antibodies onto latex spheres of 2 different sizes to enhance the accuracy and sensitivity of CRP detection. Furthermore, it seeks to establish a robust methodological framework crucial for advancing the development of latex-enhanced immunoturbidimetric detection reagents. **Introduction:** CRP, an acute-phase protein, rapidly elevates in response to infections or tissue damage. Double-particle latex-enhanced immunoturbidimetry offers important advantages for accurately measuring CRP levels. **Methods:** CRP antibodies were coupled with 2 sizes of polystyrene latex spheres. Coupling rates were evaluated to determine optimal conditions. Particle sizes suitable for CRP detection, as well as coupling and mixing ratios, were optimized using automated biochemical analysis. Transmission electron microscopy and nanoparticle size analysis were employed to characterize the morphology and size changes of CRP antibodies and coupled latex spheres before and after immune reaction. **Results:** Optimization identified 168- and 80-nm latex sphere sizes, with CRP antibody coupling rates of 92% and 91%, respectively. The optimal ratios were 10:1.5 for large latex spheres to polyclonal antibodies and 5:1.5 for small latex spheres to monoclonal antibodies. A 1:8 mixing ratio of large to small latex spheres was effective. Transmission electron microscopy confirmed uniform sizes postcoupling, maintaining dispersion with no morphological changes. CRP reacted with the double-particle latex reagent, forming immune complexes that exhibited agglutination. Mixed latex spheres showed varied agglutination states with CRP concentration, altering solution absorbance. **Conclusion:** This study validates the efficacy of the dual-particle-size CRP antibody latex reagent, highlighting its potential for future immunoturbidimetric analysis applications.

## Introduction

C-reactive protein (CRP) is a major acute-phase protein that rapidly increases in plasma during infections or tissue damage. It plays a crucial role in innate immunity by activating complements and enhancing phagocytosis [1,2]. Following acute inflammatory conditions such as tissue injuries, myocardial infarction, surgical trauma, or radiation injury, CRP levels sharply increase within hours. In severe inflammation, CRP levels can rise over a thousandfold, directly correlating with the severity of infection and injury. Besides serving as a nonspecific marker of inflammation, CRP actively participates in inflammatory processes and is linked to cardiovascular diseases like atherosclerosis [3,4]. It is considered one of the strongest predictive factors for cardiovascular diseases, including coronary artery disease and myocardial infarction [5,6]. Given its clinical value, CRP serves as a crucial biomarker in the early diagnosis of inflammatory conditions, cardiovascular risk assessment, and patient prognosis. By continuously monitoring CRP levels, healthcare professionals can quickly detect changes in disease progression, allowing them to implement targeted interventions.

Latex-enhanced immunoturbidimetric utilizes latex spheres as markers in an immunological technique based on antigen–antibody reactions. Coated with antibodies, the latex spheres interact with specific analytes, and particle aggregation quantifies analyte concentration via turbidimetry [7–9]. In latex-enhanced immunoturbidimetry, carrier latex spheres are polystyrene latex spheres of varying diameters conjugated with protein molecules. Analyte concentrations are determined by measuring light transmission reductions at specific wavelengths using a spectrophotometer [10–12]. This method offers several marked advantages, including high sensitivity, operational simplicity, and rapid detection. These characteristics make it particularly well suited for large-scale clinical testing. Additionally, it shows considerable potential for the diagnosis and monitoring of various diseases, thereby establishing itself as a reliable and efficient diagnostic tool.

The intensity of light scattering is proportional to the radius of latex spheres, allowing larger latex spheres to enhance the sensitivity of immunoturbidimetric assays. However, this increase in particle size complicates the background signal, potentially leading to interference during detection. In instances of high analyte concentrations, the detection signal may approach the upper limit of the instrument’s photometric range [13], resulting in saturation. To mitigate this issue, sample dilution is often necessary, which adds complexity to the pre-treatment process and can reduce testing efficiency. In contrast, smaller latex spheres produce less signal amplification, contributing to lower sensitivity. Nevertheless, they perform better in high-analyte-concentration scenarios due to their lower background signal generation. In summary, optimizing the assay’s dynamic range and detection limits remains a technical challenge, requiring careful adjustments of latex sphere size proportions and the selection of targeting molecules with varying affinities.

In this study, CRP antibodies were chemically coupled to carboxylated polystyrene latex spheres of 2 different sizes. Transmission electron microscopy (TEM) and nanoparticle size analysis were utilized to observe and compare the surface aggregation changes of CRP antibodies before and after coupling with latex and following immunoreaction with CRP. The coupling of CRP antibodies with dual-sized latex spheres and their reaction with CRP were confirmed by the evaluation of particle size and microdistribution. This study presents an innovative approach through the development of a dual-particle size latex immunoturbidimetric method, which enhances sensitivity and specificity in the quantification of CRP. By investigating optimal particle size combinations, we have established a robust methodological foundation that improves the precision of CRP detection and broadens the applications of this technology in laboratory diagnostics. Furthermore, this research offers new insights into the detection of other biomarkers, thereby advancing the application of latex immunoturbidimetry in clinical settings.

## Results

### Optimization of latex sphere sizes

**Fig. 1.**
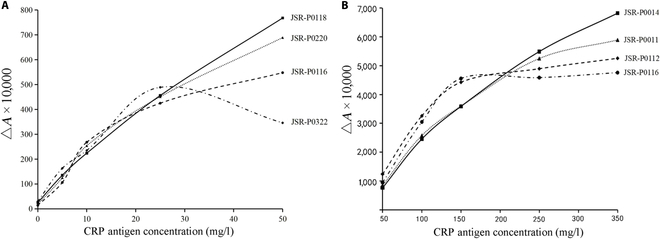
Changes in the absorbance of CRP latex spheres. (A) Large latex spheres. (B) Small latex spheres.

Large latex spheres were confirmed to enhance assay sensitivity. As shown in Table 1 and Fig. 1A, the immunolatex reagents JSR-P0116 and JSR-P0322 exhibited poor linear relationships (*R*^2^ < 0.95) with calibration standards. In contrast, JSR-P0118-immobilized latex spheres demonstrated the greatest absorbance difference (Δ*A*) variation and excellent linearity (*R*^2^ = 0.9914) within the CRP concentration range of 0 to 50 mg/l. Therefore, JSR-P0118 (168 nm) was selected as the optimal large latex sphere for this experiment.

**Table 1.** Linear fit between CRP immunolatex reagents of different particle sizes and calibration standards

Particle size category	Latex sphere	Particle size	Fitting formula	*R* ^2^
Large latex spheres	JSR-P0116	113 nm	*y* = 10.268*x* + 87.37	0.8871
	JSR-P0118	168 nm	*y* = 14.583*x* + 59.10	0.9914
	JSR-P0220	218 nm	*y* = 13.021*x* + 73.03	0.9659
	JSR-P0322	280 nm	*y* = 6.108*x* + 142.66	0.4964
Small latex spheres	JSR-P0014	80 nm	*y* = 19.649*x* + 287.59	0.9730
	JSR-P0011	94 nm	*y* = 16.269*x* + 705.29	0.9280
	JSR-P0116	113 nm	*y* = 10.787*x* + 1,638.80	0.6404
	JSR-P0112	141 nm	*y* = 11.747*x* + 1,700.50	0.7587

CRP, C-reactive protein

Small latex spheres were confirmed to extend the detection linear range. As shown in Table 1 and Fig. 1B, immunolatex reagents JSR-P0011, JSR-P0116, and JSR-P0112 displayed poor linear correlations (*R*^2^ < 0.95) with calibration standards. Among these, JSR-P0014 exhibited the maximum Δ*A* variation at CRP concentrations of 50 to 350 mg/l and the best linearity (*R*^2^ = 0.9730). Thus, JSR-P0014 (80 nm) was chosen as the optimal small latex sphere.

As shown in Table 1 and Fig. 1, JSR-P0011, JSR-P0116, and JSR-P0112 showed poor linear correlation (*R*^2^ < 0.95) with calibration standards. In contrast, JSR-P0014 exhibited the highest Δ*A* variation and the best linearity (*R*^2^ = 0.9730) within the CRP concentration range of 50 to 350 mg/l. Therefore, JSR-P0014 (80 nm) was selected for this research.

### Optimization of the antibody-to-latex-sphere concentration ratio

#### Determination of large latex sphere and antibody ratios

**Fig. 2.**
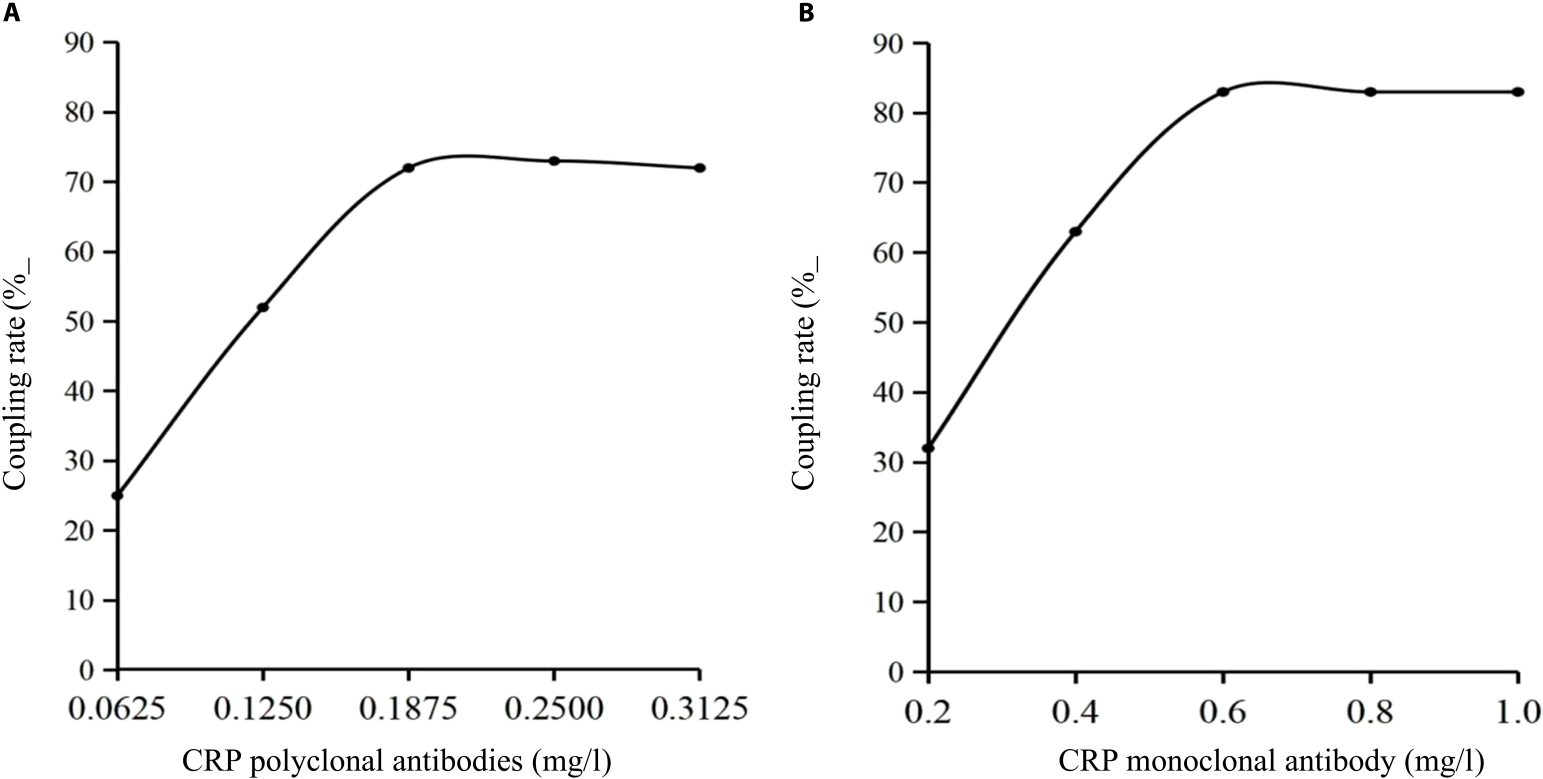
The effect of CRP antibody concentration on the coupling rate of latex spheres. (A) Large latex spheres. (B) Small latex spheres.

A 10-ml reaction system was set up using JSR-P0118 (168 nm) carboxyl-functionalized polystyrene latex spheres (solid content 100 mg/ml), adding 0.125 ml to achieve a latex sphere concentration of 1.25 mg/ml. The latex sphere ratios of 10:0.5, 10:1, 10:1.5, 10:2, and 10:2.5 corresponded to polyclonal antibodies concentrations of 0.0625, 0.125, 0.1875, 0.25, and 0.3125 mg/ml, respectively. Absorbance was measured before and after conjugation to calculate coupling efficiency Fig. 2A). For CRP polyclonal antibody concentrations below 0.1875 mg/ml, latex spheres were in excess; saturation was reached with concentrations exceeding 0.1875 mg/ml, showing a plateau in coupling efficiency with increasing antibody concentration up to 0.2500 mg/ml.

#### Determination of small latex sphere and antibody ratios

Similarly, a 10-ml reaction system was prepared using JSR-P-0014 (80 nm) carboxyl-functionalized polystyrene latex spheres (solid content 50 mg/ml), adding 0.4 ml to achieve a latex sphere concentration of 2 mg/ml. The latex sphere ratios of 5:0.5, 5:1, 5:1.5, 5:2, and 5:2.5 corresponded to monoclonal antibodies concentrations of 0.2, 0.4, 0.6, 0.8, and 1.0 mg/ml, respectively. Absorbance was measured before and after conjugation to calculate coupling efficiency (Fig. 2B).

For CRP monoclonal antibody concentrations below 0.6 mg/ml, latex spheres were in excess; saturation was reached with concentrations exceeding 0.6 mg/ml, showing a plateau in coupling efficiency up to 0.8 mg/ml. This indicated saturation of carboxyl groups on latex sphere surfaces with CRP monoclonal antibodies at concentrations of 0.1875 mg/ml for polyclonal antibodies and 0.6 mg/ml for monoclonal antibodies.

**Fig. 3.**
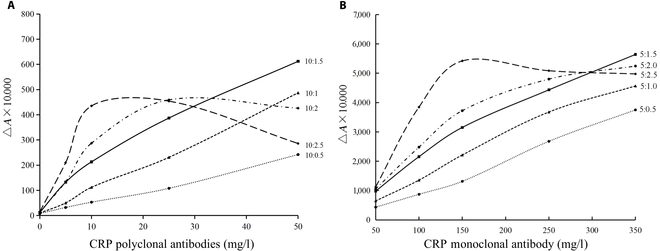
Changes in the absorbance of latex-sphere-to-CRP-antibody ratio. (A) Large latex spheres. (B) Small latex spheres.

Immunolatex reagents prepared at different latex-sphere-to-antibody ratios were tested against calibrators, as detailed in Table 2 (Fig. 3A and B). For large-diameter latex spheres coupled with polyclonal antibodies, ratios below 10:1.5 exhibited a strong linear correlation (*R*^2^ > 0.95), with increasing absorbance values enhancing sensitivity. However, further increases in ratio reduced linearity, consistent with earlier conjugation efficiency findings, confirming an optimal ratio of 10:1.5 for large-diameter latex spheres and CRP polyclonal antibodies. Similarly, for small-diameter latex spheres coupled with monoclonal antibodies, ratios below 5:1.5 showed a robust linear correlation (*R*^2^ > 0.95), expanding the linear range with higher ratios. However, excessive ratios led to reduced absorbance in highly concentrated samples, aligning with conjugation efficiency results, establishing an optimal ratio of 5:1.5 for CRP small-diameter latex spheres and CRP monoclonal antibodies.

**Table 2.** The ratio and linear fitting of spheres with different particle sizes and antibody coupling

Particle size category	Ratio of latex spheres and antibodies	Fitting formula	*R* ^2^
Large latex spheres	Latex spheres:polyclonal antibodies = 10:0.5	*y* = 4.611*x* + 5.601	0.9937
	Latex spheres:polyclonal antibodies = 10:1.0	*y* = 9.494*x* + 7.110	0.9977
	Latex spheres:polyclonal antibodies = 10:1.5	*y* = 11.446*x* + 65.172	0.9729
	Latex spheres:polyclonal antibodies = 10:2.0	*y* = 7.691*x* + 125.750	0.6710
	Latex spheres:polyclonal antibodies = 10:2.5	*y* = 3.589*x* + 215.400	0.1622
Small latex spheres	Latex spheres:monoclonal antibodies = 5:0.5	*y* = 11.370*x* − 236.140	0.9946
	Latex spheres:monoclonal antibodies = 5:1.0	*y* = 13.361*x* + 87.707	0.9887
	Latex spheres:monoclonal antibodies = 5:1.5	*y* = 15.084*x* + 558.220	0.9795
	Latex spheres:monoclonal antibodies = 5:2.0	*y* = 13.455*x* + 1,039.600	0.8902
	Latex spheres:monoclonal antibodies = 5:2.5	*y* = 10.100*x* + 2,273.400	0.4769
	Latex spheres:monoclonal antibodies = 5:0.5	*y* = 11.370*x* − 236.140	0.9946

### Optimization of antibody and latex sphere conjugation conditions

Appropriate conjugation conditions were screened using an orthogonal experimental design table (Table 3) for CRP antibodies with large and small latex spheres.

**Table 3.** Orthogonal experimental design of various factors for the coupling of CRP antibodies with large- and small-particle-size latex spheres

Number	Large latex spheres				Small latex spheres			
	Activator (mg/l)	pH of coupling buffer	Sealing agent (g/l)	Coupling rate (%)	Activator (mg/l)	pH of coupling buffer	Sealing agent (g/l)	Coupling rate (%)
1	70(1)	7.0(1)	4(2)	58	150(1)	7.0(1)	15(2)	91
2	80(2)	7.0(1)	3(1)	75	200(2)	7.0(1)	10(1)	68
3	90(3)	7.0(1)	5(3)	56	250(3)	7.0(1)	20(3)	72
4	70(1)	7.5(2)	3(1)	69	150(1)	7.5(2)	10(1)	84
5	80(2)	7.5(2)	5(3)	85	200(2)	7.5(2)	20(3)	59
6	90(3)	7.5(2)	4(2)	89	250(3)	7.5(2)	15(2)	69
7	70(1)	8.0(3)	5(3)	64	150(1)	8.0(3)	20(3)	71
8	80(2)	8.0(3)	4(2)	79	200(2)	8.0(3)	15(2)	82
9	90(3)	8.0(3)	3(1)	69	250(3)	8.0(3)	10(1)	57
*K*_1_	191	189	213		246	231	209	
*K*_2_	239	243	226		209	212	242	
*K*_3_	214	212	205		198	210	202	
*k*_1_	63.7	63.0	71.0		82.0	77.0	69.7	
*k*_2_	79.7	81.0	75.3		69.7	70.7	80.7	
*k*_3_	71.3	70.7	68.3		66.0	70.0	67.3	
Optimal choice	A_2_	B_2_	C_2_		A_1_	B_1_	C_2_	
Optimal combination	A_2_B_2_C_2_				A_1_B_1_C_2_			

Based on the results from Table 3, the optimal combination for large latex spheres (A_2_B_2_C_2_) included 80 mg/l activator concentration, pH 7.5 conjugation buffer, and 4 g/l blocking agent. For small latex spheres (A_1_B_1_C_2_), the optimal combination included 150 mg/l activator concentration, pH 7.0 conjugation buffer, and 15 g/l blocking agent, resulting in coupling efficiencies of 92% and 91%, respectively.

### Analysis of CRP immunolatex characterization

#### TEM characterization of CRP antibody coupling with latex spheres

**Fig. 4.**
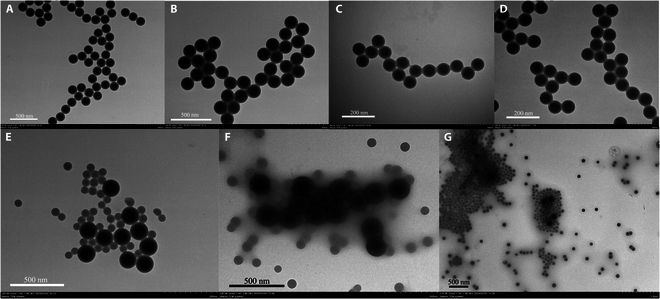
Results of latex spheres under different CRP states in transmission electron microscopy (TEM). (A) Large latex sphere bare balls. (B) Large immunolatex after coupling with antibodies. (C) Small latex sphere bare balls. (D) Small immunolatex after coupling with antibodies. (E) Mixture of immunolatex after coupling large and small latex spheres with antibodies. (F) Latex after reacting with low-concentration CRP. (G) Latex after reacting with high-concentration CRP.

Large and small latex spheres, both uncoated and separately conjugated with CRP polyclonal and monoclonal antibodies, were observed using TEM, as depicted in Fig. 4.

TEM analysis revealed that large latex spheres exhibited excellent dispersion and no aggregation prior to conjugation. Following binding with CRP polyclonal antibodies, the shape and distribution of the latex spheres remained unchanged, indicating that the conjugation process did not adversely affect their physical properties and stability. Similarly, small latex spheres displayed uniform dispersion characteristics, with no substantial alterations in appearance or arrangement after conjugation with CRP monoclonal antibodies, affirming the stability of the antibody–latex sphere conjugation process. When mixtures of latex spheres conjugated with antibodies of different sizes were combined, the resulting immunolatex maintained the original uniform distribution of both latex sphere types. This combination not only enhanced the sensitivity of CRP detection but also broadened the assay’s linear detection range.

In reactions with low CRP concentrations, large latex spheres primarily formed immune complexes (latex–CRP antibody–CRP antigen–CRP antibody–latex), while small latex spheres remained relatively dispersed without excessive aggregation. Conversely, at higher CRP concentrations, small latex spheres predominantly bound with the antigen to form immune complexes. This observation supports the theoretical strategy of using large latex conjugated with highly reactive antibodies to maximize detection sensitivity at low analyte concentrations. Conversely, the increasing involvement of small latex spheres conjugated with antibodies at higher analyte concentrations expands the detection linear range of the assay.

#### Nanoparticle size analysis characterization of CRP antibody coupling with latex spheres

**Fig. 5.**
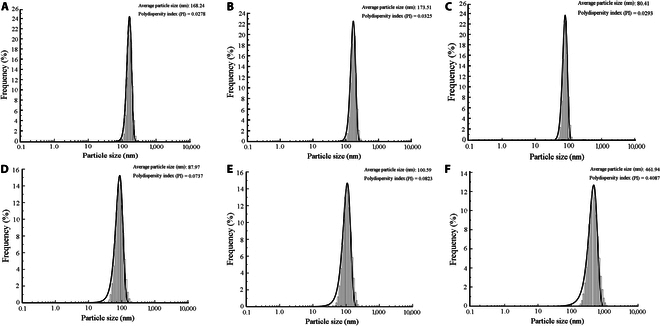
Latex sphere nanoparticle size analyzer under different CRP states. (A) Large-particle-size latex sphere bare balls. (B) Large-particle-size immunolatex after coupling with antibodies. (C) Small-particle-size latex sphere bare balls. (D) Small-particle-size immunolatex after coupling with antibodies. (E) Large- and small-particle-size latex spheres mixed in proportion after coupling. (F) Latex after reacting with CRP antigen.

Given the smaller volume of CRP antibodies compared to that of latex spheres, this experiment utilized nanoparticle size analysis before and after coupling CRP antibodies with latex spheres to evaluate antibody conjugation efficiency. The study investigated changes in the particle size distribution of both large- and small-diameter latex spheres following coupling with CRP polyclonal and monoclonal antibodies, detailed in Fig. 5A to D.

Figure 5 illustrates the particle size and distribution of latex spheres under varying CRP conditions. For large-diameter latex spheres, sizes increased from 168.24 nm (bare) to 173.51 nm after coupling with CRP polyclonal antibodies. Conversely, small-diameter latex spheres showed an increase from 80.41 to 87.97 nm after coupling with CRP monoclonal antibodies, indicating a slight size increase upon antibody surface coupling. When mixed in a 1:8 ratio, the average particle size of these latexes was 100.59 nm (Fig. 5E), reflecting a heterogeneous dispersion system where size represents the average of all latex spheres detected. The polydispersity index (PI) values of these latex sphere groups ranged from 0.0278 to 0.0823, indicating uniformly stable solutions.

Upon reaction of the mixed latex with the antigen, a latex sphere–CRP antibody–CRP antigen–CRP antibody–latex sphere immunocomplex formed, markedly increasing the particle size to 461.94 nm (Fig. 5F). This increase suggests the formation of numerous complexes postreaction, resulting in a less uniform and stable system with a higher PI value of 0.4087. These nanoparticle size analyzer results were validated by TEM findings.

## Discussion

Latex spheres, due to their ease of production, uniform size distribution, and high sensitivity, are widely utilized in quantitatively analyzing and diagnosing biomolecules in diverse biological fluids [14]. The establishment of a dual-sized latex-enhanced immunoturbidimetric assay hinges critically on the preparation of immunolatex, influenced by several key factors [15], including coupling buffer, activator and blocker dosages, latex sphere size, and the ratio of antibodies to latex spheres.

In the interaction between CRP and immunolatex spheres, the intensity of immune reactions inversely correlates with particle size. During the reaction, larger latex spheres aggregate and smaller latex spheres subsequently aggregate. In this study, CRP antibodies were coupled with latex spheres of different sizes, selecting JSR-P0118 (168 nm) for larger CRP latex spheres and JSR-P0014 (80 nm) for smaller ones. Optimization of latex sphere size showed that larger spheres exhibited a steeper slope in the regression curve for samples with low CRP concentrations, thereby enhancing analytical sensitivity. Smaller latex spheres demonstrated increasing absorbance with rising concentrations of high CRP samples, improving measurement linearity. The combination of large and small latex spheres ensures both high sensitivity and a broader linear range.

Immunoreactivity decreases with increasing particle size and charge [12,16]. Theoretically, smaller latex spheres offer larger surface areas, requiring more antibodies for labeling, whereas larger spheres, with smaller surface areas, require fewer antibodies for labeling [12]. As confirmed by this study, when the concentration of large-particle CRP antibodies is 0.1875 mg/ml and that of small-particle CRP antibodies is 0.6 mg/ml, the reaction between the carboxyl groups on the surface of the latex spheres and the antibodies reaches saturation. Furthermore, the coupling ratio of small latex spheres to CRP antibodies is 5:1.5. In contrast, the coupling ratio of large latex spheres to CRP antibodies demonstrates good performance when it is less than 10:1.5, indicating effective antigen–antibody interactions.

Based on the Derjaguin–Landau–Verwey–Overbeek theory [17], the stability of latex is influenced by both electrostatic and steric repulsion. Electrostatic repulsion strength is closely linked to the surface charge density of the latex particles and the electrolyte concentration in the aqueous phase, such as pH. In contrast, steric repulsion is provided by partially water-soluble oligomers or polymers. Research [18] indicates that under a fixed electrolyte background, the zeta potential shifts from negative to positive with increasing amounts of papain, resulting in a U-shaped stability curve. The lowest stability is observed at the point of charge neutralization, after which stability gradually increases. This phenomenon is explained by the Derjaguin–Landau–Verwey–Overbeek theory, which considers the dynamic interactions between colloidal particles and oppositely charged substances, such as multivalent ions. Therefore, the selection of activators, the pH of coupling buffers, and the choice of blocking agents are crucial for the stability of antibody coupling and latex reagents. Inappropriate coupling conditions may lead to reaching the critical coagulation concentration, resulting in the aggregation and sedimentation of colloidal particles. This study systematically evaluated the coupling conditions. For larger latex particles, the optimal combination identified was A_2_B_2_C_2_, which includes 80 mg/l activator, a coupling buffer at pH 7.5, and 4 g/l blocking agent. Under these conditions, no marked aggregation of the latex reagents was observed, and stability was markedly enhanced.

This study characterizes the latex spheres before and after antibody coupling, as well as their interaction with antigens to form immune complexes. TEM images illustrate that prior to antibody coupling, latex spheres exhibit a uniform size and stable particle spacing without aggregation. After antibody coupling, there is no substantial alteration in latex sphere morphology or distribution, indicating that this process does not affect particle dispersion. Electron microscopy clearly reveals pronounced aggregation among latex spheres upon reaction with CRP, resulting in the formation of immune complexes. This biological phenomenon, where antigen–antibody interactions are quantified as physical signals, is analyzed using Lambert–Beer’s law to determine antigen concentrations. TEM images demonstrate the aggregation behavior of dual-sized immunolatex spheres when mixed with samples of varying CRP concentrations, consistent with previous studies [19]. Larger latex spheres aggregate at lower analyte concentrations, whereas smaller latex spheres aggregate at higher concentrations. These findings validate TEM’s effectiveness in characterizing the physical interactions and aggregation dynamics of immune complexes formed during antigen–antibody reactions.

Nanoparticle size analysis reveals that the average diameter of large latex spheres increased from 168.24 to 173.51 nm following coupling with polyclonal antibodies, and that of small latex spheres increased from 80.41 to 87.97 nm following coupling with monoclonal antibodies, confirming successful antibody immobilization on latex sphere surfaces. The dispersion coefficients of these latex spheres were 0.0278, 0.0325, 0.0293, and 0.0737, respectively, indicating low polydispersity (PI values) and demonstrating the uniformity and stability of singly coupled antibody latex spheres. When mixed at a ratio of 1:8, the resulting mixture had an average diameter of 100.59 nm (PI = 0.0823). Upon reaction with CRP, the latex sphere immune complexes exhibited an average diameter of 461.94 nm (PI = 0.4087), indicating marked changes in diameter and PI. The increase in diameter and PI suggests the formation of more complexes in the solution after the CRP reaction, correlating with changes in photoelectric signals. The results of the nanoparticle size analysis and the TEM showed mutually confirmed results, which were consistent with the previous research results [20].

## Conclusion

This study confirms the effective application of dual-sized latex spheres in CRP immunoassays. By integrating both large and small latex spheres, enhanced detection capabilities were achieved, allowing for accurate quantification of CRP levels. Characterization through electron microscopy and nanoparticle size analysis validated the successful immobilization of antibodies and the formation of immune complexes. These findings provide valuable insights for advancing latex-based immunoassay methods, highlighting their potential for effective biomolecular detection in biomedical research.

## Materials and Methods

### Instruments and reagents

The nanoparticle size analyzer was obtained from Jinan Micro Nano Technology Co., Ltd. (China). An HT7800 transmission electron microscope and a Hitachi 7100 fully automatic biochemical analyzer were purchased from Hitachi (Japan). A Shimadzu 2800 ultraviolet (UV) spectrophotometer was purchased from Shimadzu Instruments Co., Ltd. (China). 1-Ethyl-(3-dimethylaminopropyl) carbodiimide hydrochloride (EDC·HCl; 98%) and *N*-hydroxysuccinimide (NHS) were purchased from Aladdin. CRP rabbit antihuman polyclonal antibodies (14.91 mg/ml), CRP mouse antihuman monoclonal antibodies (12.7 mg/ml), and CRP antigen (500 mg/l) were purchased from Nanjing KeyGen Biotech. Polystyrene latex spheres were purchased from JSR Corporation (Japan). Bovine serum albumin was purchased from Roche (Switzerland). 2-Morpholinoethanesulfonic acid (MES) was purchased from Suzhou Yacoo Science Co., Ltd. (China). An artificial serum matrix was purchased from Shenzhen Braveds (China). The other chemical reagents were purchased from Sinopharm Chemical Reagent Co., Ltd. (China).

### Calibration standards

CRP antigens with a concentration of 500 mg/l were diluted in purified water to prepare calibration standards at 0, 5, 10, 25, 50, 100, 150, 250, and 350 mg/l.

### Preparation and optimization of latex reagents

#### Preparation of coupling process solutions

Sample dilution solution R1 was prepared by dissolving 2.2897 g of Na_2_HPO_3_, 0.6039 g of NaH_2_PO_3_·2H_2_O, and 12.5 g of NaCl in 1 l of purified water, achieving a pH of 7.20. This solution was stored at 2 to 8 °C. For the activation buffer, 9.762 g of MES was dissolved in 1 l of purified water, and the pH was adjusted to 5.5 using 4 M NaOH, resulting in a 50 mmol/l MES solution. Similarly, the coupling buffer was prepared by dissolving 1.9625 g of Na_2_HPO_3_ and 0.9625 g of NaH_2_PO_3_·2H_2_O in 1 l of purified water to reach a pH of 7.30, and it was stored under identical conditions. The blocking buffer, designed to prevent nonspecific binding, consisted of 100 g of bovine serum albumin, 1.9625 g of Na_2_HPO_3_, and 0.9625 g of NaH_2_PO_3_·2H_2_O in 1 l of purified water adjusted to pH 7.30; it was also stored at 2 to 8 °C. Lastly, the storage buffer was prepared by dissolving 6.057 g of Tris in 1 l of purified water, adjusting the pH to 8.00 using 8 mmol/l HCl, and stored under refrigeration. All chemicals used were of analytical grade.

#### Coupling of latex spheres with CRP antibodies

To prepare CRP immunolatex solutions, an activation buffer is mixed with large and small latex spheres (80 to 280 nm) in a 1:1 ratio, along with EDC·HCl and NHS solutions. This mixture is incubated at 37 °C with shaking at 220 rpm for 30 min and then centrifuged at 18,000 rpm and 4 °C for 60 min to obtain a precipitate, which is resuspended in coupling buffer and sonicated for 5 min. CRP monoclonal antibodies are added, and the solution is incubated at 37 °C with shaking at 220 rpm for 90 min. The volume of antibody added (*V*_CRP_) is calculated using *V*_CRP_ = (*m*_CRP_/*C*_CRP_), where *m*_CRP_ is the mass of CRP antibodies and *C*_CRP_ denotes the concentration of CRP antibodies.

After antibody coupling, a blocking buffer is added, and the solution is incubated at 37 °C with shaking at 220 rpm for 60 min. The mixture is centrifuged again at 18,000 rpm and 4 °C for 20 min, and the supernatant is discarded. For aging, the precipitate is transferred to a storage buffer, sonicated for 30 min, and aged in a 37 °C incubator for 72 h to yield CRP immunolatex solution A. The entire procedure is repeated using large latex spheres and CRP polyclonal antibodies to obtain CRP immunolatex solution B. Finally, solutions A and B are mixed in a predetermined ratio to produce CRP immunolatex solution R2.

#### UV absorption method for coupling efficiency

The coupling efficiency of antibodies to latex spheres is assessed using a UV spectrophotometer. First, the absorbance of the blank coupling solution is measured at 280 nm (denoted as OD_0_). Subsequently, precoupled antibodies are mixed with the coupling solution, and the absorbance of this mixture is measured (denoted as OD_1_). Following the centrifugation of the postcoupled antibody and latex sphere complexes, the absorbance of the supernatant is measured (denoted as OD_2_). The coupling efficiency is then calculated using the following formula: $\text{coupling efficiency}=\left[\left({\mathrm{OD}}_{1}-{\mathrm{OD}}_{2}\right)/{\mathrm{OD}}_{0}\right]\times 100\%$. In this formula, OD_0_ represents the absorbance of the coupling buffer, OD_1_ indicates the absorbance of the antibodies prior to coupling, and OD_2_ corresponds to the absorbance of antibodies that did not participate in the reaction, as measured in the supernatant after coupling.

#### Optimization of the reaction system

The initial parameters of the biochemical analyzer are set according to Table S1. In summary, the sample is incubated with the diluent (R1) for 5 min, then the immunolatex solution (R2) is added, and the absorbance value (*A*_0_) is measured at this point. After that, the sample is incubated for an additional 5 min and the absorbance (*A*_1_) is measured. The CRP concentration is calculated based on the difference in absorbance (Δ*A* = *A*_1_ − *A*_0_) and the reaction curve.

#### Optimization of the CRP latex sphere size

According to clinical diagnostic requirements (linear detection range up to 350 mg/l and detection limit of 0.1 mg/l), the latex sphere size is optimized for linear correlation and detection limit. It is optimized within the range of 100 to 280 nm for large spheres and 80 to 145 nm for small latex spheres. The particle size of latex spheres was selected as the single influencing factor, and latex spheres of different particle sizes were coupled with CRP antibodies to complete the preparation of immunolatex solution.

Different latex sphere sizes are experimentally screened for their individual impact on CRP detection. Large spheres conjugated with CRP polyclonal antibodies are expected to detect lower concentrations of CRP samples, using calibration standards of 0 to 50 mg/l to calculate Δ*A* and the linear correlation coefficient (*R*^2^). Small latex spheres conjugated with CRP monoclonal antibodies are expected to detect higher concentrations of CRP samples, using calibration standards of 50 to 350 mg/l to calculate the absorbance difference (Δ*A*) and linear correlation coefficient (*R*^2^). The optimal latex sphere size is selected based on the calibration results.

#### Optimization of the antibody-to-latex-sphere ratio for CRP

Equation 1 is used to estimate the required antibody amount:

$$S=\frac{6}{\rho d}\left(C\right)$$ (1)

In the formula, *S* is the amount of antibodies bound to the surface of latex spheres, *ρ* is the density of latex spheres, *d* is the diameter of latex spheres, and *C* is the binding constant of antibodies on the surface of latex spheres. The above formula can be used to preliminarily estimate the amount of antibodies used, and the coupling effect of antibodies and latex spheres can be evaluated by the coupling rate.

The antibody-to-latex-sphere coupling efficiency is evaluated using coupling efficiency. CRP detection reagents are prepared using different ratios of latex spheres to CRP antibodies (large latex spheres:antibodies 10:0.5, 10:1, 10:1.5, 10:2, and 10:2.5; small latex spheres:antibodies 5:0.5, 5:1, 5:1.5, 5:2, and 5:2.5). These reagents are tested with known concentrations of CRP calibration standards. The optimal latex-to-antibody ratio is selected based on the absorbance difference (Δ*A*) versus CRP calibration curve, coupling efficiency, and linear range.

#### Optimization of antibody conjugation conditions with latex spheres

The concentration of the activator (EDC·HCl/NHS), the pH of the conjugation buffer, and the amount of blocking agent exhibit marked interactions that impact the conjugation efficiency of antibodies with latex spheres. These factors were selected as orthogonal experimental factors, each with 3 levels, to conduct a 4-factor 3-level orthogonal experiment. Conjugation efficiency was evaluated as the criterion to identify the optimal conjugation process for antibodies with latex spheres. The factors and levels of the CRP orthogonal experiment design are presented in the orthogonal experiment. Statistical analysis was performed using the SPSSAU system.

## Data Availability

The original contributions presented in the study are included in the article and the Supplementary Materials; further inquiries can be directed to the corresponding authors.
