# Supplementary material for: Preparation and Characterization of C-Reactive Protein Dual-Particle Latex-Enhanced Immunoturbidimetric Reagents
Source: BME Front. 2024 Dec 23;5:0085. doi: 10.34133/bmef.0085 (PMC11665802; doi:10.34133/bmef.0085)
Supplement: Supplementary 1 — Fig. S1 Table S1 [file bmef.0085.f1.zip › Table S1.docx]

Table S1. Parameters of CRP fully automatic biochemical analyzer

| Name | parameter |
| --- | --- |
| Main wavelength | 570nm |
| Sub wavelength | 700nm |
| Calibration method | Six point calibration |
| Calibration type | Logit-Log 5P |
| Reaction direction | Positive reaction |
