# Supplementary figures and images for: Preparation and Characterization of C-Reactive Protein Dual-Particle Latex-Enhanced Immunoturbidimetric Reagents
Source: BME Front. 2024 Dec 23;5:0085. doi: 10.34133/bmef.0085 (PMC11665802; doi:10.34133/bmef.0085)

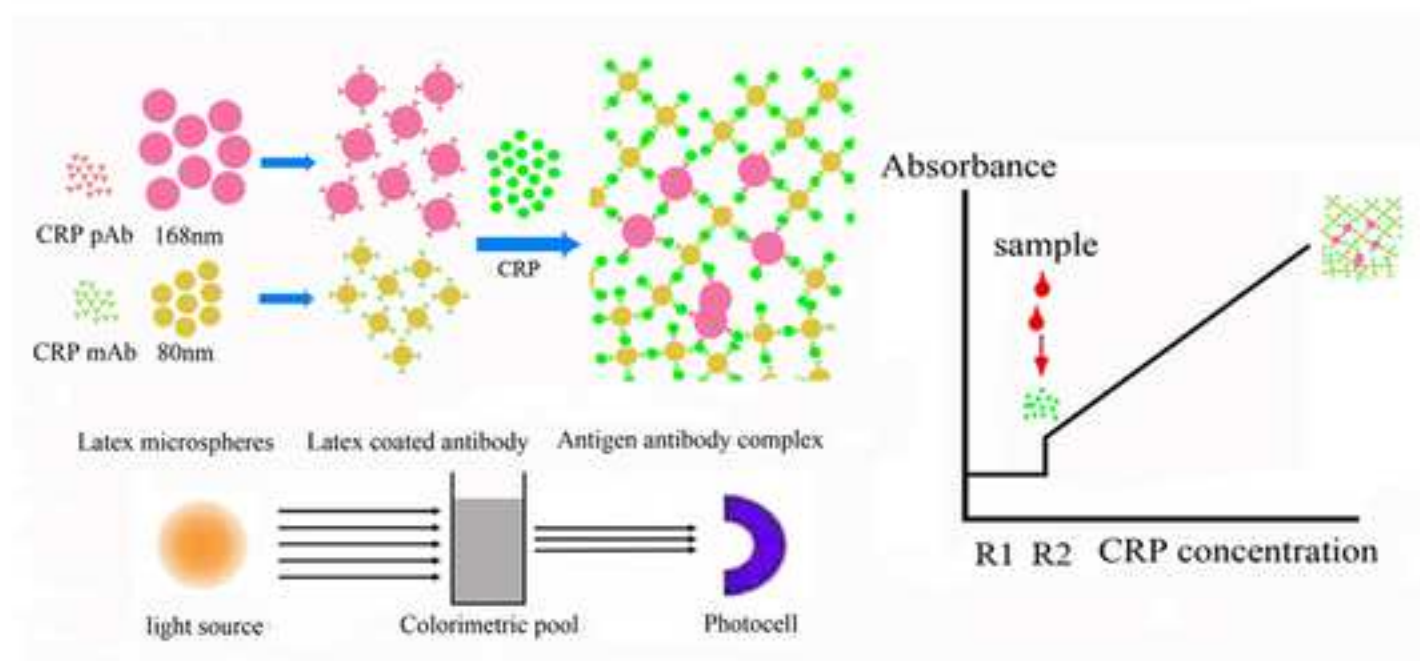

Fig. S1 The preparation process and the mechanism of CRP detection immune reaction.

Supplement: Supplementary 1 — Fig. S1 Table S1 [file bmef.0085.f1.zip › BMEF-D-24-00035.pdf]
